# *Hydraena* (s.str.) *dinarica*, new species (Coleoptera: Hydraenidae) along with further records of *Hydraena* spp. from Durmitor National Park, Montenegro and comments on the DNA barcoding problem with the genus

**DOI:** 10.3897/BDJ.9.e59892

**Published:** 2021-01-12

**Authors:** Hendrik Freitag, Rick de Vries, Marta Paterno, Simone Maestri, Massimo Delledonne, Cameron G Thompson, Helena Lamed, Rebekah Lambert, Michael F Fox, Mariela C Gonzalez, Emmanuel D Delocado, Marc R Sabordo, Clister V Pangantihon, Iva Njunjić

**Affiliations:** 1 Ateneo de Manila University, Quezon City, Philippines Ateneo de Manila University Quezon City Philippines; 2 Amstelveenseweg 980B, Amsterdam, Netherlands Amstelveenseweg 980B Amsterdam Netherlands; 3 University of Verona, Department of Biotechnology, Strada Le Grazie 15, Verona, Italy University of Verona, Department of Biotechnology, Strada Le Grazie 15 Verona Italy; 4 Oxford Brookes University, Department of Health and Life Sciences, Oxford, United Kingdom Oxford Brookes University, Department of Health and Life Sciences Oxford United Kingdom; 5 39 av Melbourne, Mont-Royal, Quebec, Canada 39 av Melbourne, Mont-Royal Quebec Canada; 6 340 N. Adams Street, Eugene, Oregon, United States of America 340 N. Adams Street Eugene, Oregon United States of America; 7 Taxon Expeditions, Rembrandtstraat 20, Leiden, Netherlands Taxon Expeditions, Rembrandtstraat 20 Leiden Netherlands; 8 Naturalis Biodiversity Center, Darwinweg 2, Leiden, Netherlands Naturalis Biodiversity Center, Darwinweg 2 Leiden Netherlands

**Keywords:** citizen science, MinION sequencing, minute moss beetle, Palaearctic region, Taxon Expeditions, Durmitor Mt.

## Abstract

**Background:**

Long-palped Water Beetles were collected during a taxon expedition in Montenegro which involved citizen scientists, students and taxonomists. The material was collected from springs, brooks, fens and the Tara River, at altitudes between 600 m and 1450 m above sea level, using fine-meshed hand-nets and by manual checking of submerged substrates. The morphological species delimitation was supplemented and congruent with mtDNA sequences mainly obtained in the field using the newly-developed MinION-based *ONTrack* pipeline.

**New information:**

The new species *Hydraena dinarica* Freitag & de Vries, sp. n. from Durmitor Mt. is described, illustrated and compared in detail to closely-related congeners of the *H. saga* d'Orchymont, 1930/*H. emarginata* Rey, 1885 species complex. Five additional species and female specimens of two unidentified morphospecies of the genus were also recorded in the vicinity of Durmitor National Park. New records and the first DNA barcodes for *Hydraena biltoni* Jäch & Díaz, 2012 (endemic to Montenegro) and *H. morio* Kiesenwetter, 1849 are provided. Further records of *H. nigrita* Germar, 1824, *H. minutissima* Stephens, 1829, *H. subintegra* Ganglbauer, 1901 and females of two unidentified morphospecies are commented upon. The resulting inter- and intraspecific genetic distances and some observations of low or zero sequence divergence between recently-diverged species of *Hydraena* Kugelann, 1794 are briefly discussed.

## Introduction

The Long-palped Water Beetles of the genus *Hydraena*, originally described by Kugelann (1794), represent the most speciose aquatic coleopteran genus. In tropical Asia, where the genus is still under-explored, a new species has even been discovered in the middle of a megacity (Freitag 2013). Nineteen *Hydraena* species are currently recorded from Montenegro. Many species of the genus are endemic to comparably small distribution ranges (Jäch et al. 2005), such as *H. biltoni* Jäch & Díaz, 2012 and *H. latebricola* Jäch, 1986 in Montenegro (see Jäch 1986, Jäch and Díaz 2012). The Mediterranean area is particularly diverse and new species are still being discovered (e.g. Bilton 2013, Mičetić Stanković and Jäch 2012, Jäch and Díaz 2012). Therefore, the first author targeted *Hydraena* when instructing a citizen scientist project during a “taxon expedition” to Montenegro in 2019. The principles and great benefit of such initiatives are discussed by Schilthuizen et al. (2017) and Freitag et al. (2018). Through the enthusiastic support of citizen scientists, several aquatic habitats in and around Durmitor National Park, a UNESCO world heritage site, were sampled for aquatic beetles. The variety of collection sites included springs, creeks, lakes and fens in forests and alpine meadows, up to the stunning torrent of the Tara River in Europe's deepest gorge.

**Figure 1.**
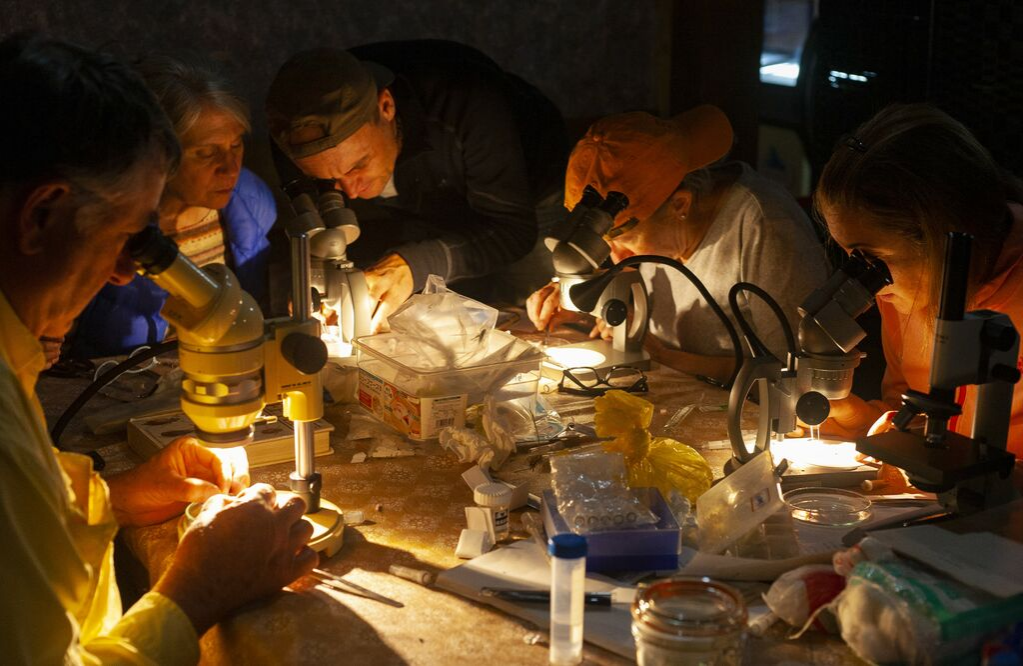
Citizen scientists working in an improvised lab during the taxon expedition to Durmitor.

**Figure 2.**
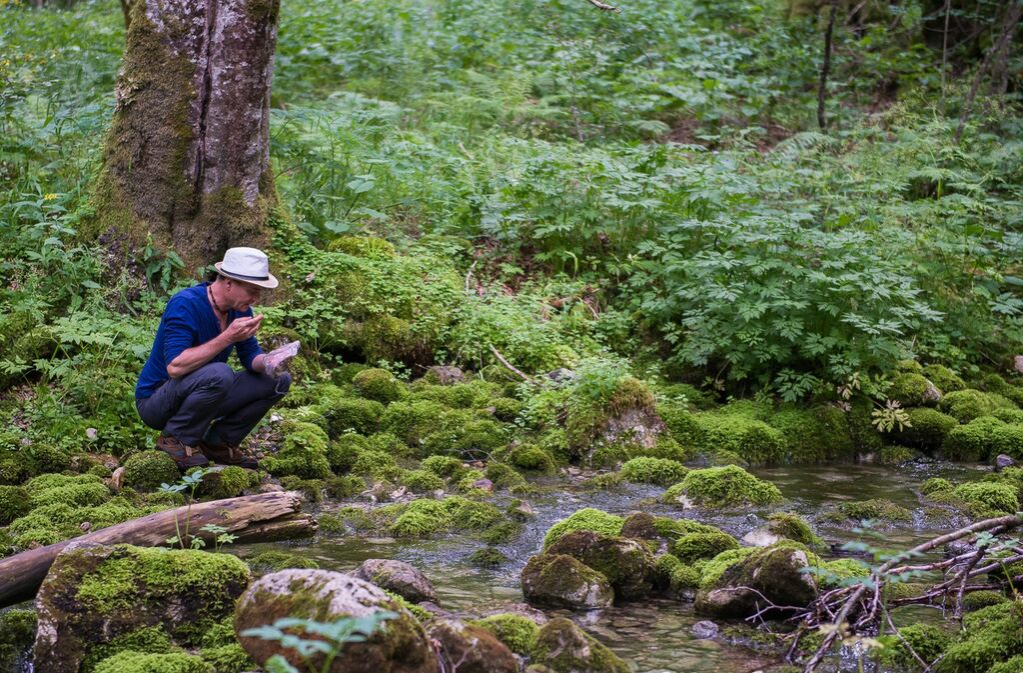
Skakala stream at the northern slopes of Durmitor massif, type locality of *Hydraena dinarica*, sp. n.

The subsequent identification of the collected material at the improvised field laboratory (Fig. 1, Suppl. material 2), set up in a holiday resort “Etno selo Šljeme” near the town of Žabljak, revealed eight *Hydraena* (s.str.) species, amongst which one new species was actually discovered. The new species was found at Skakala stream, a mountain creek flowing from Skakala waterfall into the periodically-inundated Sušica Lake on the northern slopes of Durmitor massif (Fig. 2).

## Materials and methods

Specimens were collected in a microhabitat-specific approach (Freitag 2015) by disturbing the submerged substrates of the water body and collecting any floating specimens using fine-meshed hand-nets. Larger solid substrates (e.g. submerged wood) were taken off from the water and checked for specimens. The material was immediately preserved in vials with 96% ethanol, separately for each microhabitat assessed.

Pre-sorting and genus-level identification were performed by taxon expedition participants mentored by the first author (Fig. 1), using taxonomic literature and dissection microscopes. Specimens of *Hydraena* were then dissected by the help of fine pincers and entomological pins to reveal their most diagnostic sexual characters (aedeagus, gonocoxite and tergite X) and compared with descriptions of *Hydraena* species of the Balkan Region. Their genitalia, temporarily mounted in lactic acid on microscopic slides, were examined under a Leica ICC50 HD compound microscope.

Detailed examination and digital imaging of dissected parts was done using an Olympus CX21 microscope equipped with a DinoEye Eyepiece camera. Habitus photographs were taken under a Zeiss Axio Zoom V 16 microscope with a Canon 5D Mark II SLR attached to the microscope. Images were captured at various focus planes and subsequently stacked using the Helicon Focus software. Genital drawings were compiled after their photographs by vector graphic tools in CorelDRAW v.10.0 software, but in direct comparison with the actual genitals mounted on slides.

After removal of diagnostic parts (glued on entomological cards), the entire remaining specimen of each initially-recognised morphospecies and some unidentifiable female specimens underwent DNA isolation, amplification, sequencing and processing of the 5′-end of the mitochondrial cytochrome oxidase I (*COI*) gene as described in Maestri et al. (2019) (see https://github.com/MaestSi/ONTrack). The primer pair LCO1490 and HC02198 was used for PCR amplification. Library preparation for the MinION Oxford Nanopore NGS device was performed using SQK-LSK109 (Run1) and SQK-LSK108 (Run2) kits and, for each library, samples were pooled together after adding index sequences. The final libraries were loaded on a R9.4.1 Flongle flow-cell (Run1) and on a R9.4.1 MinION flow-cell (Run2). Sequencing was carried out in the field using an off-line version of MinKNOW v1.6.11. The two sequencing runs were stopped after 5 and 17 hours and produced a total of 182,504 and 533,919 sequence reads, respectively. Sequence reads were base-called and demultiplexed using Guppy v.3.1.5 and accurate consensus sequences were generated using the *ONTrack* pipeline v.1.2.2 (Maestri et al. 2019). After our return from the field expedition, some additional sequences were generated by conventional Sanger sequencing by a commercial service using PCR products amplified as described above and following standard protocols. Forward and reverse Sanger reads were manually assembled into a consensus sequence using BIOEDIT version 7.2.5 (Hall 1999).

The DNA sequence alignment, which also included available standard barcodes of related *Hydraena* species of the region of Hendrich et al. (2015), Pentinsaari et al. (2014), Ribera et al. (2011), Rulik et al. (2017), Trizzino et al. (2011) and Trizzino et al. (2013b), was manually generated with the software MEGA X (Kumar et al. 2018).

A statistical parsimony haplotype network was constructed by TCS1.21 (Clement et al. 2002), visualised using POPART (Leigh and Bryant 2015) and further edited in Adobe Illustrator 2020. The genetic sequence divergence analysis was performed in MEGA X using Kimura-2-parameter (K2P) model with the bootstrap method in 1000 replicates.

DNA sequences were submitted to International Nucleotide Sequence Database Collaboration (INSDC) through GenBank, as well as to the Barcode of Life Data System (BOLD) under project TXEX.

The type labels of the new species are literally quoted from the specimen’s label under 'bibliographicCitation'. Back slashes indicate the next line in the label.

## Taxon treatments

### Hydraena (Hydraena) biltoni

Jäch & Díaz, 2012

DF236B2B-BE4D-5E2D-A313-63F640388761

#### Materials

- **Type status:** Other material. **Occurrence:** recordedBy: Hendrik Freitag, Michael F. Fox, Rebekah Lambert; individualID: H69; sex: 3 males, 2 females; lifeStage: adults; associatedSequences: GenBank: MT784158.1; **Location:** locationID: MNE21c; continent: Europe; waterBody: small Black Lake tributary; country: MONTENEGRO; municipality: Žabljak; locality: Durmitor National Park; verbatimLocality: small Black Lake tributary creek near war monument, pine forest, pebble in shallow run; verbatimElevation: 1435 m; verbatimCoordinates: 43 08 57N; 19 05 42E; **Identification:** identifiedBy: Hendrik Freitag, Rick De Vries, Cameron G. Thompson, Helena Lamed, Rebekah Lambert, Michael F. Fox, Mariela Gonzalez, Clister V. Pangantihon; **Event:** eventDate: 2019-07-16; **Record Level:** institutionCode: CFM, ZMB; collectionCode: Coleoptera; basisOfRecord: Dried specimens; informationWithheld: MONTENEGRO: Žabljak, Durmitor N.P., small Black Lake tributary creek near war monument, pine forest, pebble in shallow run, 1435 m a.s.l., 43 08 57N 19 05 42E, 16 July 2019, leg M.F. Fox, R. Lambert, H. Freitag (MNE21c)

#### Distribution

**Figure 3.**
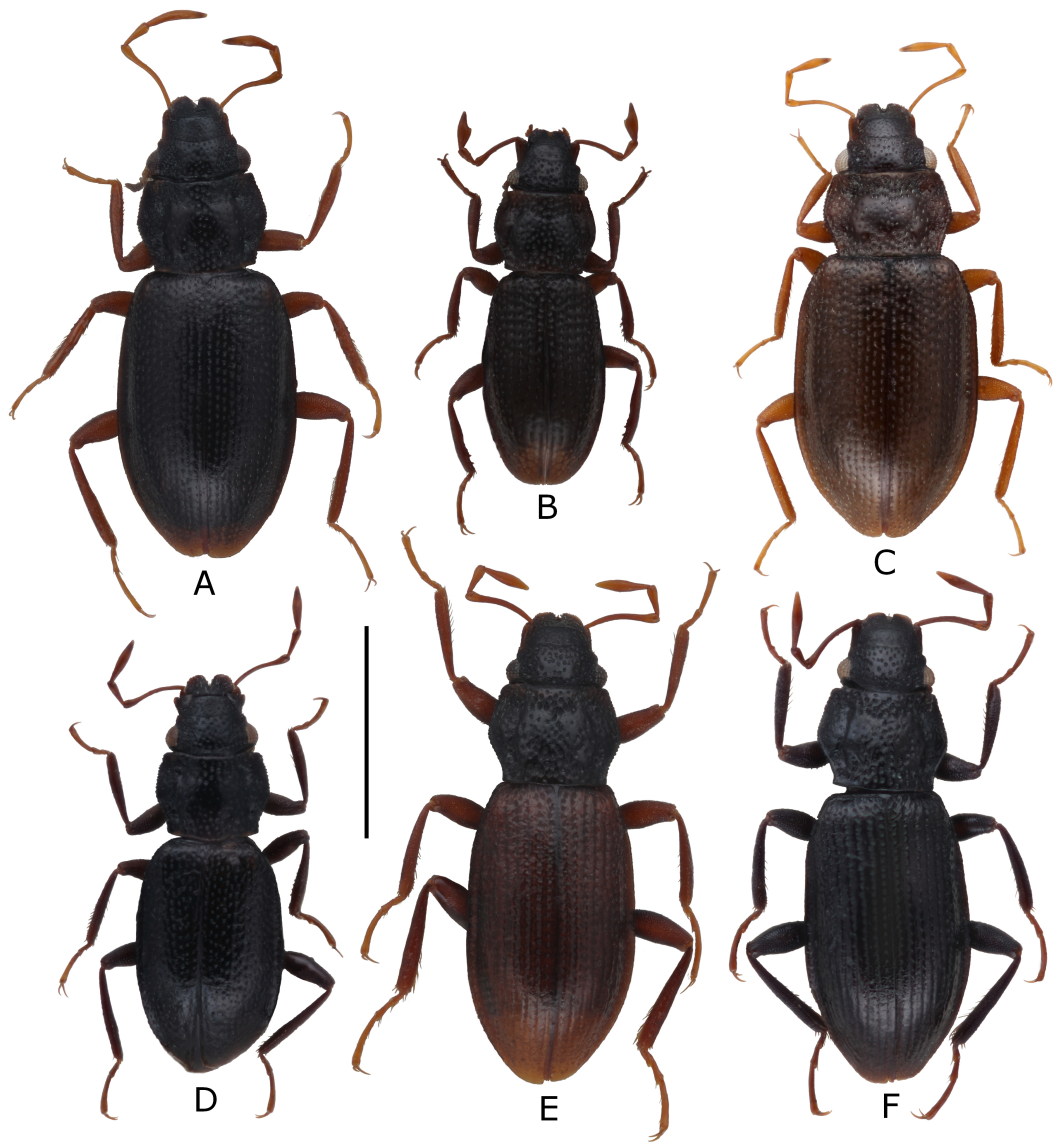
Digital habitus photograph of A) *Hydraena biltoni* ♂; B) *H. minutissima* ♂; C) *H. morio* ♀; D) *H. nigrita* ♂; E) *H. subintegra*, pale colouration of a teneral ♂ specimen; F) *H. subintegra* ♂, regular colouration; scale bar = 1.0 mm.

*Hydraena biltoni* (Fig. 3A) is endemic to Montenegro. Previously, it was collected from the vicinity of Šavnik, about 20 km south of Žabljak and Biogradska Gora (Jäch and Díaz 2012).

#### Remarks

We provide the first standard barcode for the species. It varies by only 0.3% from that of the closest congener *H. morio* Kiesenwetter, 1849 (Suppl. material 1). As *H. biltoni* is extremely similar to the latter, which also occurs in the region, thorough examination of the aedeagus is required for proper identification.

#### Habitat

The specimens were collected in a very small creek, flowing through pine forest and a wet meadow. Bottom pebbles, mixed with CPOM, in moderately fast flowing, shallow portions of the creek, were their microhabitat.

### Hydraena (Hydraena) minutissima

Stephens, 1829

902BD376-8719-5BD7-A622-98D1E96D89E2

#### Materials

- **Type status:** Other material. **Occurrence:** recordedBy: Hendrik Freitag, Clister V. Pangantihon; individualID: H60; sex: 2 males, 5 females; lifeStage: adults; associatedSequences: GenBank: MT784159.; **Location:** locationID: MNE17b; continent: Europe; waterBody: Shallow littoral, pool with pebbles; country: Montenegro; municipality: Žabljak; locality: Tara River; verbatimLocality: Tara River, near Bijela Stijena, shallow littoral pool with pebbles; verbatimElevation: 600 m; verbatimCoordinates: 43 13 23N; 19 09 57E; **Identification:** identifiedBy: Hendrik Freitag, Rick De Vries, Cameron G. Thompson, Helena Lamed, Rebekah Lambert, Michael F. Fox, Mariela Gonzalez, Clister V. Pangantihon; **Event:** eventDate: 2019-07-12; **Record Level:** institutionCode: CFM, ZMB; collectionCode: Coleoptera; basisOfRecord: Dried specimens

#### Distribution

*Hydraena minutissima* (Fig. 3B), originally described from Great Britain (Stephens 1829), is widely distributed in southern, western and central Europe from Spain and Turkey in the south up to the British Isles (Jäch and Skale 2015).

#### Habitat

The species was collected in the shallow littoral of the Tara River with pebble deposits on bedrock. The microhabitat was not exposed to strong currents during the time of collection and filamentous algae were partly growing on the surrounding exposed rocks.

### Hydraena morio

Kiesenwetter, 1849

912A7FCA-4ABC-5EE1-A0E3-C7C3FA11AE3D

#### Materials

- **Type status:** Other material. **Occurrence:** recordedBy: Hendrik Freitag, Clister V. Pangantihon; individualID: H62, H64, H70, H71; sex: 7 males, 2 females; lifeStage: adults; associatedSequences: GenBank: MT784154.1, MT784155.1, MT784156.1, MT784157.1; **Location:** locationID: MNE17b; continent: Europe; waterBody: Small littoral pool with pebbles; country: Montenegro; municipality: Žabljak; locality: Tara River; verbatimLocality: Tara River, near Bijela Stijena, shallow littoral pool with pebbles; verbatimElevation: 600 m; verbatimCoordinates: 43 13 23N; 19 09 57E; **Identification:** identifiedBy: Hendrik Freitag, Rick De Vries, Cameron G. Thompson, Helena Lamed, Rebekah Lambert, Michael F. Fox, Mariela Gonzalez, Clister V. Pangantihon; **Event:** eventDate: 2019-07-12; **Record Level:** collectionCode: Coleoptera; basisOfRecord: Dried specimen

#### Distribution

*Hydraena morio* (Fig. 3C) is mainly distributed in eastern and central Europe in an area from Turkey to Germany, including the Balkan Region (Jäch and Skale 2015).

#### Remarks

We provide here the first *COI* 5′-end sequences (Folmer Region) of the species. See also remarks on *H. biltoni.*

#### Habitat

For notes on the habitat, see *Hydraena minutissima*.

### Hydraena (Hydraena) nigrita

Germar, 1824

2CF23352-129D-5B96-960E-9BAA24F23DAE

#### Materials

- **Type status:** Other material. **Occurrence:** recordedBy: Hendrik Freitag, Clister V. Pangantihon; individualID: H61; sex: 2 males; lifeStage: adults; associatedSequences: GenBank: MT784150.1; **Location:** locationID: MNE17b; continent: Europe; waterBody: Shallow littoral pool with pebbles; country: Montenegro; municipality: Žabljak; locality: Tara River; verbatimLocality: Tara River, near Bijela Stijena, shallow littoral pool with pebbles; verbatimElevation: 600 m; verbatimCoordinates: 43 13 23N; 19 09 57E; **Identification:** identifiedBy: Hendrik Freitag, Rick De Vries, Cameron G. Thompson, Helena Lamed, Rebekah Lambert, Michael F. Fox, Mariela Gonzalez, Clister V. Pangantiho; **Event:** eventDate: 2019-07-12; **Record Level:** institutionCode: CFM, ZMB; collectionCode: Coleoptera; basisOfRecord: Dried specimen

#### Distribution

*Hydraena nigrita* (Fig. 3D), originally described from Germany (Germar 1824), is distributed from Greece and Eastern Europe up to the British Isles (Jäch and Skale 2015).

#### Habitat

For notes on the habitat, see *Hydraena minutissima*.

### Hydraena (Hydraena) spp.

693187E8-F5E3-583D-9030-88477ABA7EF7

#### Materials

- **Type status:** Other material. **Occurrence:** recordedBy: Hendrik Freitag; individualID: H65; sex: 3 females; lifeStage: adults; associatedSequences: GenBank: MT784151.1; **Location:** locationID: MNE10l; continent: Europe; waterBody: Fen meadow near Durmitor N.P entrance; country: Montenegro; municipality: Žabljak; locality: Durmitor N.P.; verbatimLocality: Fen meadow near Durmitor N.P entrance, littoral pool; verbatimElevation: 1425 m; verbatimCoordinates: 43 06 08N; 19 10 56E; **Identification:** identifiedBy: Hendrik Freitag, Rick De Vries, Cameron G. Thompson, Helena Lamed, Rebekah Lambert, Michael F. Fox, Mariela Gonzalez, Clister V. Pangantihon; identificationRemarks: “cf. *britteni*”; **Event:** eventDate: 2019-07-10; **Record Level:** institutionCode: CFM; collectionCode: Coleoptera; basisOfRecord: Dried specimen
- **Type status:** Other material. **Occurrence:** recordedBy: Hendrik Freitag; individualID: H66; sex: 2 females; lifeStage: adults; associatedSequences: GenBank: MT784152.1; **Location:** locationID: MNE13k; continent: Europe; waterBody: fen meadow near Dobri Nugo; country: Montenegro; municipality: Žabljak; locality: Dobri Nugo; verbatimLocality: Fen meadow near Dobri Nugo, partly subterranean water flow, slow run; verbatimElevation: 1392 m; verbatimCoordinates: 43 09 15N; 19 06 06E; **Identification:** identifiedBy: Hendrik Freitag, Rick De Vries, Cameron G. Thompson, Helena Lamed, Rebekah Lambert, Michael F. Fox, Mariela Gonzalez, Clister V. Pangantihon; identificationRemarks: "sp. female"; **Event:** eventDate: 2019-07-11; **Record Level:** institutionCode: CFM; collectionCode: Coleoptera; basisOfRecord: Dried specimen

#### Remarks

The samples cluster with *Hydraena britteni* Joy, 1907, originally described from England and Ireland (Joy 1907), vary only by ca. 0.3% and 2.5%, respectively, in their genetic distance from the latter. Based on the known distribution range of *H. britteni*, which does not include Montenegro and the genetic distance, it remains uncertain if either specimens are conspecific with the latter. Due to the lack of male specimens, we currently cannot identify these specimens with certainty.

#### Habitat

The specimens were collected from fen-like meadows, one (MNE10) densely vegetated with sedge and horsetail, the other (MNE13) additionally with limestone boulders and gravel densely covered with mosses. In both sites, a creek with clear brownish water, rich in humins, was passing the fens and provides continuous water inputs.

### Hydraena subintegra

Ganglbauer, 1901

F252DDF2-5B04-5405-8EAC-BDC4770C4A7F

#### Materials

- **Type status:** Other material. **Occurrence:** recordedBy: Hendrik Freitag & Clister Pangantihon; individualID: H63; sex: 1 male, 1 female; lifeStage: adults; associatedSequences: GenBank: MT784149.; **Location:** locationID: MNE17c; continent: Europe; waterBody: Black Lake tributary creek; country: Montenegro; municipality: Žabljak; locality: Durmitor N.P.; verbatimLocality: Tara River near Bijela Stijena, littoral, run with pebble; verbatimElevation: 600 m; verbatimCoordinates: 43 13 23N; 19 09 57E; **Identification:** identifiedBy: Hendrik Freitag, Rick De Vries, Cameron G. Thompson, Helena Lamed, Rebekah Lambert, Michael F. Fox, Mariela Gonzalez, Clister V. Pangantihon; **Event:** eventDate: 2019-07-12; **Record Level:** institutionCode: CFM; collectionCode: Coleoptera; basisOfRecord: Dried specimens
- **Type status:** Other material. **Occurrence:** recordedBy: Helena Lamed, Mariela Gonzales, Rebekah Lambert, Michael F. Fox, Clister V. Pangantihon; individualID: H63; sex: 1 male, 1 female; lifeStage: adults; **Location:** locationID: MNE20c/f/h; continent: Europe; waterBody: Black Lake tributary creek; country: Montenegro; municipality: Žabljak; locality: Durmitor N.P.; verbatimLocality: Black Lake tributary creek near old watermill, pine forest; verbatimElevation: 1450 m; verbatimCoordinates: 43 09 09N; 19 05 22E; **Identification:** identifiedBy: Hendrik Freitag, Rick De Vries, Cameron G. Thompson, Helena Lamed, Rebekah Lambert, Michael F. Fox, Mariela Gonzalez, Clister V. Pangantihon; **Event:** eventDate: 2019-07-16; **Record Level:** institutionCode: NMW, ZMB; collectionCode: Coleoptera; basisOfRecord: Dried specimens

#### Distribution

The species is distributed in an area between the Adriatic and Black Seas, including the Dinaric Alps (Jäch and Skale 2015).

#### Remarks

The taxonomy of this species of the "*Haenydra*" lineage is not yet finally resolved. Three slightly varying morphs are recognised. Our specimens (Fig. 3E, F) belong morphologically and geographically to “Morph A” *sensu* Jäch and Díaz (2012). The standard DNA barcode of this morph which we are providing herein varies, in fact, by 0.5% from “Morph B” (Bulgaria).

#### Habitat

All specimens were collected from moderately fast flowing, shallow water, but on varying substrates, including submerged wood, grass bunches and pebble.

### Hydraena (Hydraena) dinarica

Freitag & de Vries sp. n.

4F3B289D-E691-5E70-92B2-14E60323E8FB

urn:lsid:zoobank.org:act:E5A0DED7-16AC-4B94-B9B1-72000874197D

TXEX049-20

#### Materials

- **Type status:** Holotype. **Occurrence:** recordedBy: Hendrik Freitag, Clister V. Pangantihon; sex: male; lifeStage: adult; **Location:** locationID: MNE18; continent: Europe; waterBody: Skakala stream; country: Montenegro; municipality: Žabljak; locality: Durmitor, Peradova gora; verbatimElevation: 1220 m; locationRemarks: cold water karst creek with predominant flow subsurface; verbatimCoordinates: 43 09 54N; 18 59 59E; **Identification:** identifiedBy: Hendrik Freitag; **Event:** samplingProtocol: Manual collection by hand-net from bottom substrates; eventDate: 2019-07-13; **Record Level:** type: Dried specimen; bibliographicCitation: MONTENEGRO: Durmitor, Peradova gora, \ Skakala stream, ca. 1220 m asl., \ 43°09′54″N 18°59′59″E, 13 July 2019 \ leg. H. Freitag & C.V. Pangantihon (MNE18); institutionCode: NMW; basisOfRecord: Dried specimen; informationWithheld: Terminal parts of abdomen, aedeagus and right foretasus (broken off) glued separately on to same entomological card along with holotype specimen.
- **Type status:** Paratype. **Occurrence:** recordedBy: Hendrik Freitag, Clister V. Pangantihon; individualID: H67, H68; sex: 14 males, 11 females; lifeStage: adults; associatedSequences: GenBank: MT784148.1; **Location:** locationID: MNE18; continent: Europe; waterBody: Skakala stream; country: Montenegro; municipality: Žabljak; locality: Durmitor, Peradova gora; verbatimElevation: 1220 m; locationRemarks: cold water karst creek with predominant flow subsurface; verbatimCoordinates: 43 09 54N; 18 59 59E; **Identification:** identifiedBy: Hendrik Freitag, Rick De Vries, Cameron G. Thompson, Helena Lamed, Rebekah Lambert, Michael F. Fox, Mariela Gonzalez, Clister V. Pangantihon; **Event:** samplingProtocol: Manual collection by hand-net from bottom substrates; eventDate: 2019-07-13; **Record Level:** type: Dried specimens; bibliographicCitation: MONTENEGRO: Durmitor, Peradova gora, \ Skakala stream, ca. 1220 m asl., \ 43°09′54″N 18°59′59″E, 13 July 2019 \ leg. H. Freitag & C.V. Pangantihon (MNE18); institutionCode: CFM, NMW, SMTD, ZMB; collectionCode: Coleoptera
- **Type status:** Paratype. **Occurrence:** recordedBy: Vladimir Pešić; sex: 1 female; lifeStage: adult; **Location:** continent: Europe; country: Montenegro; municipality: Žabljak; locality: Durmitor, Zeleni Vir; **Identification:** identifiedBy: Manfred A. Jäch; **Event:** samplingProtocol: Manual collection; eventDate: 2002-08-02; **Record Level:** type: Dried specimen; institutionCode: NMW; collectionCode: Coleoptera

#### Description

**Figure 4.**
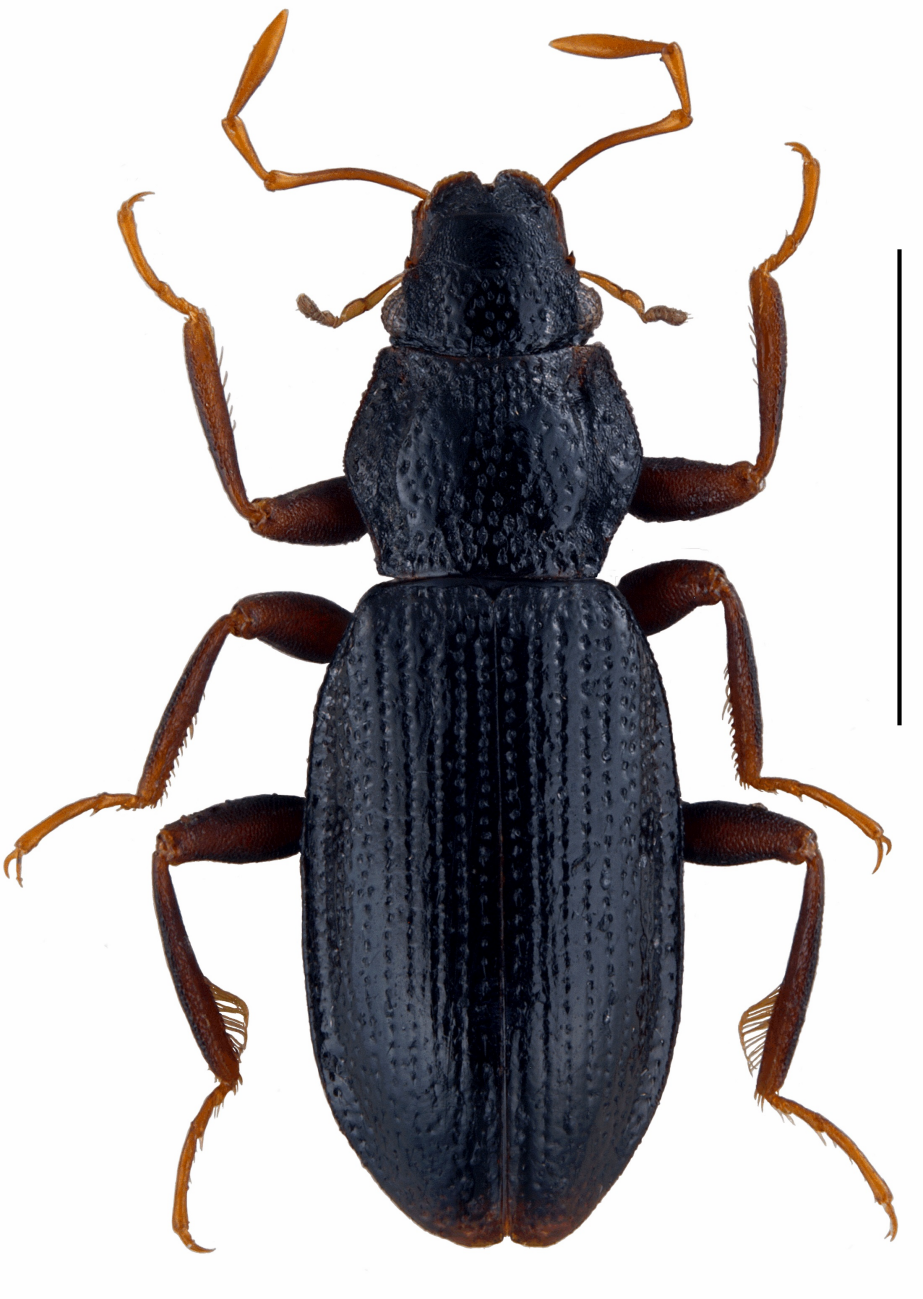
Digital habitus photographs of *Hydraena dinarica*, sp. n., paratype ♂; scale bar = 1.0 mm.

Habitus as in Fig. 4. Body (labrum to elytral apex) 2.25–2.45 mm long, 0.81–0.86 mm wide. Head, pronotum and elytra dark brown to black, femora and tibiae slightly paler dark brown, palpi and tarsi yellowish-brown. Labrum densely micropunctate, with deep anterior notch; margins slightly upturned. Clypeus medially densely micropunctate, gradually more microstriate laterad. Fronto-clypeal suture bisinuately arched, slightly impressed. Frons medially moderately densely punctate; interstices glabrous; lateral portions densely (sometimes rugosely) bipunctate; micropunctures very dense; interocular grooves indiscernible. Eyes moderately large, distinctly protruding, about 30 facets visible in dorsal view. Maxillary palpi about as long as body width.

Pronotum broadly subhexagonal, moderately wider than long; anterior and posterior margins slightly concave; anterior and posterior angles bluntly rounded, lateral rim denticulate, most conspicuous anteriorly; disc slightly convex; sagittal, anterior and posterior portions densely punctate; remaining disc portions moderately densely punctate; interstices glabrous; anterior and posterior sublateral foveae slightly impressed, rather inconspicuous; entire lateral portions slightly deflexed, rugulously bipunctate, partly microstriate.

**Figure 5.**
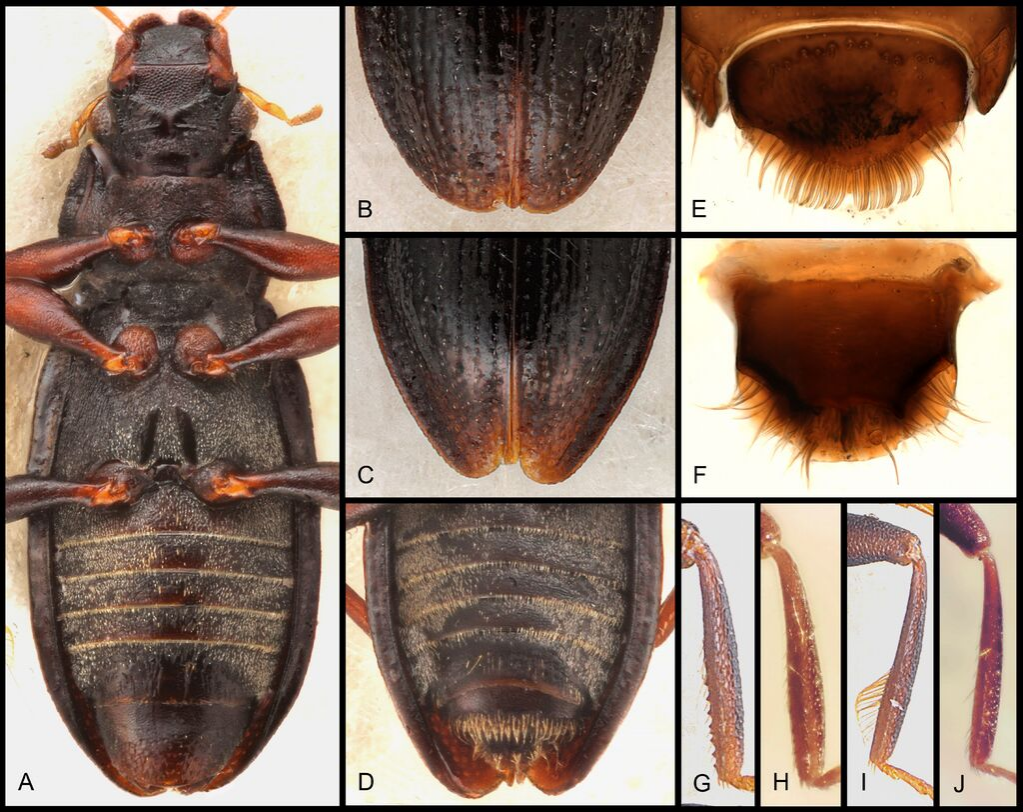
*Hydraena dinarica*, sp. n., paratypes: A) ♂ ventral view; B) ♂ elytral apices; C) ♀ elytral apices; D) ♀ ventral abdomen; E) ♀ tergite X; F) gonocoxite; G) ♂ mesotibia; H) ♀ mesotibia; I) ♂ metatibia; J) ♀ metatibia.

Elytra elongate, almost parallel-sided apical 0.15–0.70; disc slightly vaulted, sublaterally more abruptly declivitous; elytral margin moderately explanate up to ca. apical 0.15. Elytra with six regularly arranged, not or slightly impressed rows of puncture striae between suture and disc declivity (approx. at the middle of shoulder) and ca. six additional, less regular puncture striae between disc declivity and elytral margin; punctures moderately large and moderately deeply impressed on anterior disc, gradually slightly decreasing in size and degree of impression towards apex and margin; intervals and interstices flat and glabrous; intervals smaller than puncture diameter anteriorly, larger in posterior and lateral portions; apical sutural teeth present or absent, apices separately rounded, sexually dimorphic (Fig. 5B, C).

Ventral side as in Fig. 5A. Mentum and submentum densely micropunctate. Genae and gula dominantly micropunctate, partly striate; posterior genal ridge distinct, glabrous. Hypomeron micropunctate to microreticulate. Prosternum densely micropunctate with hydrofuge micropubescence, with conspicuous median keel. Mesoventrite densely micropunctate with hydrofuge micropubescence, deeply impressed anterior to mesocoxae; impression transverse-arcuate; with pair of posteriad divergent glabrous streaks lateral to mesocoxae, across mesoventral impression (rather inconspicuous in some specimens); mesoventral disc and process convex. Metaventrite densely micropunctate with hydrofuge micropubescence, central disc shallowly impressed; metaventral plaques distinct, divergent posteriad; intermetacoxal process declined posteriad, predominantly glabrous. Pseudepipleuron longitudinally impressed, with one indistinct puncture stria, rugulose and most anteriorly with hydrofuge pubescence, increasingly glabrous posteriad. Ventrites I–IV (externally visible sternites III–VI) medially flat and with moderately long pubescence (most conspicuous in young specimens), remaining portions with dense, short pubescence; ventrites V and VI largely non-pubescent, glabrous to reticulate.

Male terminal sternite subsemicircular, 0.18 mm wide, not distinguishable from *H. saga* and similar species (comp. Jäch and Díaz (2017): Figs. 5, 10 and 15); spiculum slightly curved in apical half, 0.70–0.73 mm long (vs. ca. 0.75 mm in *H. saga*; comp. Jäch and Díaz (2017): Fig. 10).

**Figure 6.**
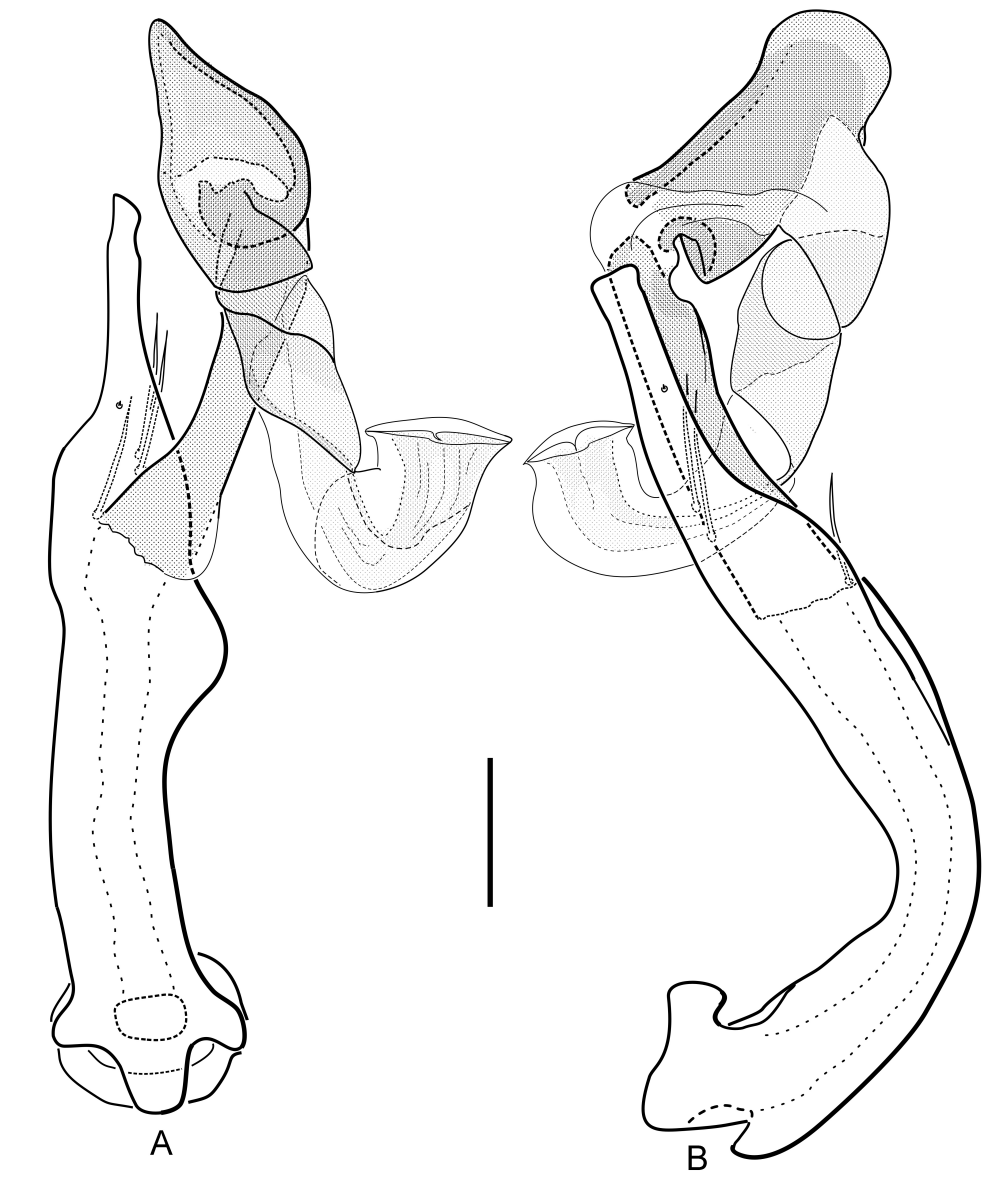
*Hydraena dinarica*, sp. n. aedeagus (holotype): A) dorsal view: B) lateral view; scale bar = 0.1 mm.

Aedeagus (Fig. 6): Total length ca. 780 μm; main piece (630 μm long) with three long setae on inner (left) side and a very short one on outer (right) side; apex somewhat variable from convex to obliquely truncate, narrowest subapically; dorsal corner very slightly produced; main piece moderately slender, basal portion rectangularly bent from apical portion, gently narrowed apical 0.25 towards slender, subparallel apical portion; right margin (dorsal view) distinctly roundly produced at about mid-length; prebasal tooth short and blunt. Phallobase subsymmetrical in dorsal and ventral views. Distal lobe generally very similar of that in *H. saga* and related species, overall more stretched than compact; submembranous contorted distal portion relatively long and wide; opening funnel-like and apicad directed (like in a tuba), located at the most right (behind main piece and distal lobe trunk in dextrolateral view, Fig. 6B); most sclerotised enlarged distal portion with conically-pointed apex in dorsal and ventral views (Fig. 6A).

Female tergite X (Fig. 5E), except for suboval shape, very similar to those of *H. saga* and related species (comp. Jäch and Díaz (2017): Figs. 7 and 12); apex widely rounded; disc with sub-basal squamose setae and with few trichoid setae; squamose setae comparably elongate and not conspicuously widened apically; subapical fringe admedially with dense fringe of vermiform setae of equal length which are slightly bent in apical half and with few long trichoid setae laterally.

Gonocoxite (Fig. 5F) very similar to those of *H. saga* and related species (comp. Jäch and Díaz (2017): Figs. 6 and 11), subtrapezoidal; hyaline apex round; inner plate moderately projecting basally and laterally; apical area densely pubescent; basal area without setae; cavity oval.

Spermatheca not examined.

Secondary sexual characters: Female elytral apices produced and separately gently rounded, not acuminate. All femora of male slightly more inflated. Male ventrite VI enlarged (Fig. 5A). Male mesotibia with a row of ca. ten denticles along proximal half of mesial face (Fig. 5G), in females only with setae (Fig. 5H). Male metatibia with fringe of long setae at inner face of posterior half (Fig. 5I), in females only with regular setae (Fig. 5J).

#### Differential Diagnosis

*Hydraena dinarica*, sp. n. is morphologically very similar to species that are referred to as *H. saga* complex (*sensu* Jäch and Díaz (2017); see Discussion), namely *H. alpicola*, *H. diazi* Trizzino, Jäch & Ribera, 2011, *H. emarginata* Rey, 1885, *H. fosterorum* Trizzino, Jäch & Ribera, 2011, *H. kahleni* Jäch & Díaz, 2017, *H. larissae* Jäch & Díaz, 2000, *H. saga* and *H. samnitica* Fiori, 1904 (original descriptions by Fiori (1904), Jäch and Díaz (2000), Jäch and Díaz (2017), Orchymont (1930a), Rey (1885), Trizzino et al. (2011a)). All of them are most typically characterised by their articulate, contorted aedeagal distal lobe and the rounded or truncate apex of the aedeagal main piece. *Hydraena belgica* d’Orchymont, 1930; *H. dalmatina* Ganglbauer, 1901; *H. hispanica* Ganglbauer, 1901; *H. pangaei* Jäch, 1992; *H. pelops* Jäch, 1995 and *H. tarvisina* (Ferro, 1991) (original descriptions by Ferro (1991), Ganglbauer (1901), Jäch (1992), Jäch (1995), Orchymont (1930b)) were additionally added by Trizzino et al. (2013a)and defined as *H. emarginata* complex (which would also include *H. lotti* Bilton, 2013 (see Bilton (2013)). They share the structural plan of the aedeagal distal lobe, but possess an acute aedeagal main piece and lack the minute seta on the outer (right) side of the main piece. Therefore, the latter species are not discussed here in detail, but it should be noted that *H. dalmatina* might occur sympatrically and is externally quite similar, but can be distinguished by the widely explanate elytral margin in apical third and its elytral apices (acuminate in males, truncate in females).

In comparison with all species mentioned above, *Hydraena dinarica*, sp. n. is unique in the tuba-like 180° bent hyaline distal tube of the aedeagus (Fig. 6) and thus upward directed opening (vs. downward or lateral directed). Its distal portion is overall larger than in all other species of the *H. saga* complex (as defined above), except for *H. emarginata*, some specimens of *H. larissae* and *H. samnitica* with subequally large distal portion. Similarly, the aedeagus of *H. dinarica*, sp. n. is larger (main piece 630 μm long) than most species mentioned above (510–610 μm), except for *H. emarginata* (610–665 μm).

Within this complex, *H. dinarica*, sp. n. seems morphologically most similar to the Italian species *H. kahleni* and *H. larissae*, especially based on their moderately large contorted aedeagal distal lobe, as well as *H. saga* on the external habitus. While *H. dinarica*, sp. n. is slightly larger (2.25–2.45 mm long) than the latter three species (1.95–2.30 mm long), the elytral disc appears slightly flatter and the elytral margin very slightly more explanate in *H. dinarica*, sp. n. The elytral apices are similar and within the observed variation range in the former species in both sexes. The new species also resembles *H. samnitica* of almost the same size (especially in the moderately large contorted aedeagal distal lobe), but it is externally distinguishable from *H. samnitica* by the explanate elytral margin extending almost up to the apex (vs. reaching apical 0.15; the apical area therefore appears more slender in *H. dinarica*, sp. n.).

**Figure 7.**
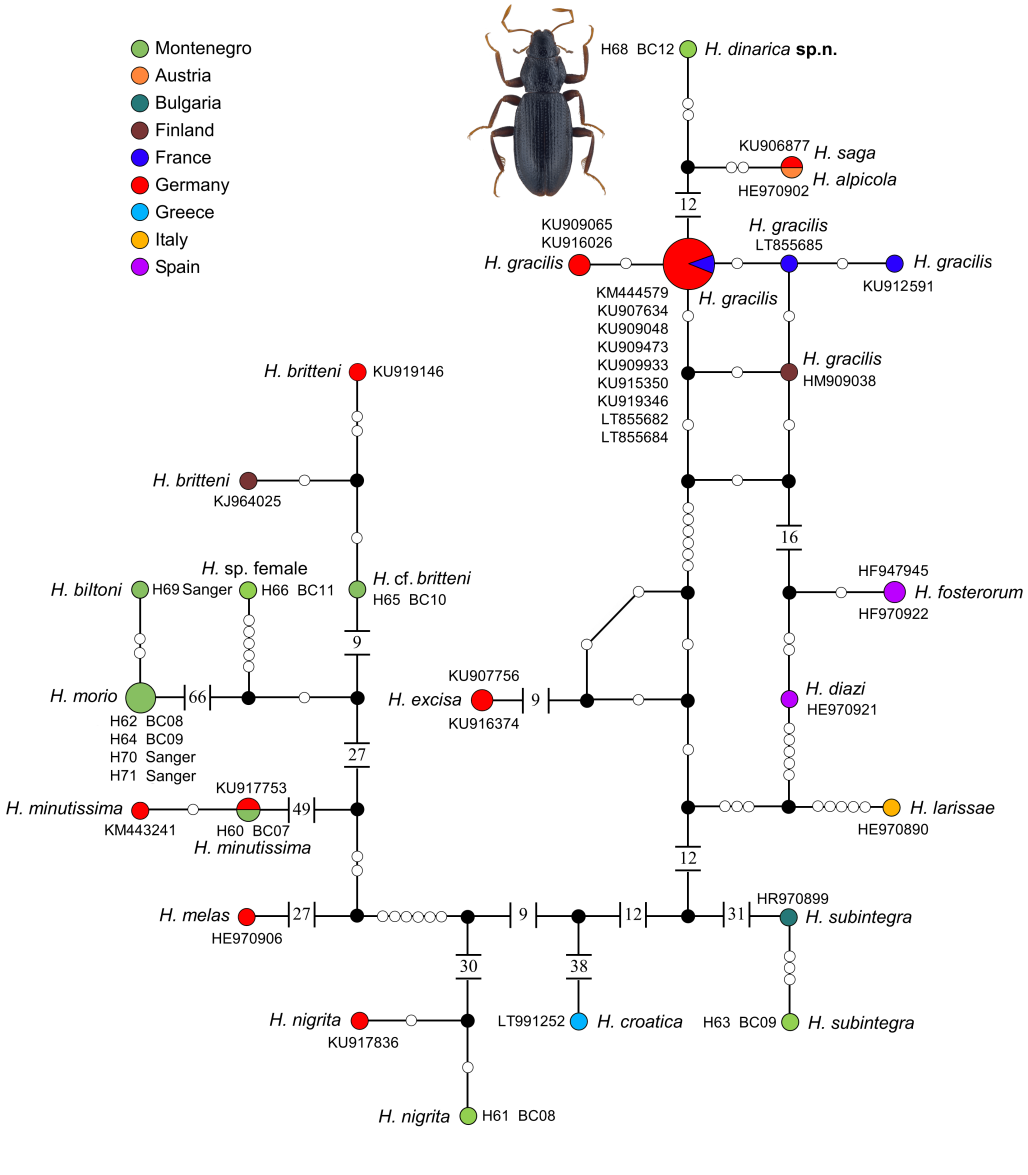
Statistical parsimony haplotype network of successfully sequenced samples of *Hydraena dinarica* sp. n., *H. biltoni, H. minutissima*, *H. morio*, *H. nigrita*, *H. subintegra*, two unidentified female specimens and GenBank records of related species from aligned *COI* sequences of 567 bp length.

On the other hand, *H. dinarica*, sp. n. seems genetically closest to *H. alpicola*, *H. saga* and the *H. gracilis* Germar, 1824 complex (as defined by Jäch (1995)), based on the DNA barcode (Fig. 7). *H. saga* occurs in the region (material at NMW; the closest known collection site is in Foča, Bosnia, less than 50 km away from the type locality of *H. dinarica*, sp. n.) and is also most similar. Therefore, the species can only reliably be identified by dissection of its aedeagus.

Males can be distinguished as stated above, while in females of *H. dinarica*, sp. n., the gonocoxite (Fig. 5F) is subtrapezoidal, with evenly round and expanded apical hyaline area (vs. subquadrate gonocoxite and with short apical area in *H. saga*; Jäch and Díaz (2017): Fig. 11). Furthermore, the female tergite X in *H. dinarica*, sp. n. (Fig. 5E) is suboval, its basal portion expanded and the vermiform setae of the subapical fringe are bent (vs. subtriangular tergit X with short basal portion and with almost straight subapical vermiform setae in *H. saga*; Jäch and Díaz (2017): Fig. 12).

Externally, the new species also resembles other representatives of the "*Haenydra*" lineage which might occur sympatrically, but differ in certain characters: *Hydraena bosnica* Apfelbeck, 1909 (posteriorly sinuate pronotum, oval elytra; Trizzino et al. (2013a): Fig. 42a); *H. excisa* Kiesenwetter, 1849 (clypeus shagrened; elytra less elongate, more oval with disc more convex, margin more explanate, apices slightly truncate in males, sharply (not roundly) notched in females; Trizzino et al. (2013a): Fig. 32l); *H. gracilis balcanica* d'Orchymont, 1930 (elytra with disc more convex, margin less explanate, apices almost conjointly rounded in males; Trizzino et al. (2013a): Fig. 24a); *H. phallica* d'Orchymont , 1930 (clypeus shagrened; elytra more oval with disc more convex, apices slightly truncate in males, shallowly notched in females; *Trizzino et al. (2013a)*: Fig. 32p); *H. subintegra* (smaller and paler, elytra reddish-brown with disc more convex, margin more explanate, apices conjointly rounded in both sexes); *H. vedrasi* d’Orchymont, 1931 (elytra with disc more convex, apex subtruncate in males, sharply (not roundly) notched in females; Trizzino et al. (2013a): Figs. 22a and g) (original descriptions by Apfelbeck (1909), Kiesenwetter (1849), Orchymont (1930a), Orchymont (1930b), Orchymont (1931)).

*Hydraena dinarica*, sp. n. varies by 0.6% genetic distance (657 bp *CO1* barcode from the most similar congeners *H. alpicola* and *H. saga* and by 3.3% from the morphologically similar *H. larissae* (Suppl. material 1).

#### Habitat

This species was collected from a mountain stream in a forested, undisturbed karst area at an altitude of about 1220 m a.s.l. (Fig. 2). During the time of collection, the river was only partly on the surface; the predominant flow was subsurface, causing low water temperatures. The well-shaded riverbed, including the water-bearing reaches, was densely covered with mosses. The specimens were collected from the upper interstitial of the bottom gravel (mesopsammon) in shallow, partly rapidly flowing water.

#### Distribution

**Figure 8.**
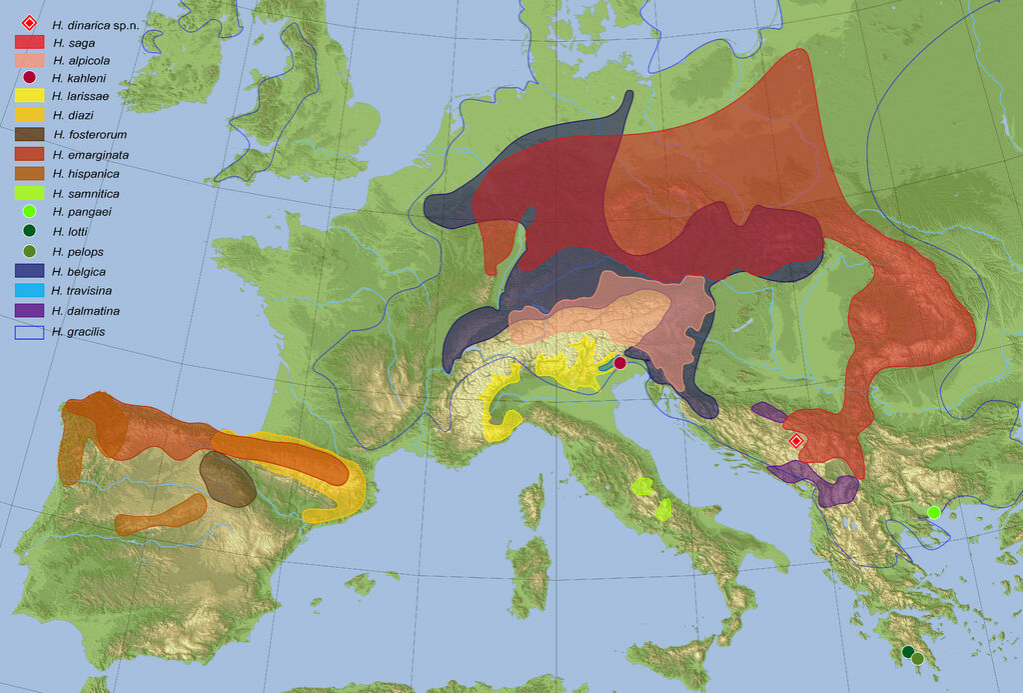
Map of Europe with the collection site of *Hydraena dinarica* sp. n. and the distribution of morphologically similar species of the "*Haenydra*" lineage defined as "*Hydraena emarginata* complex" by Trizzino et al. (2013a) and *H. gracilis* as genetically similar species with overlapping range.

So far only known from the type locality at the northern slopes of Durmitor Mt., Montenegro (Fig. 8).

#### Etymology

The species is named after the Dinaric Alps, or Dinarides, a karst mountain range where Durmitor Mt. and the type locality of the new species are situated. The epithet is used as an adjective meaning "of the Dinaric Alps".

## Analysis

### DNA barcoding

The amplification of all sequences but one (H67), with LCO1490 & HC02198 primers and the applied protocols, was successful (Table 1).

One repository sequence (KU906877), labelled "*Hydraena gracilis*", does not cluster together with the remaining samples of this species, but rather with *H. alpicola* Pretner, 1931 (a species found around the northern and eastern Alps and first recognised by Pretner (1931)), to which its barcode is identical. Given that: (1) the underlying study is a multi-taxon methodological paper testing a bioinformatic pipeline and not a taxonomic contribution (Rulik et al. 2017); (2) species of the *H. gracilis* main clade are often hardly distinguishable without dissection of the male genitalia; (3) if not exactly the same place, the collection site of the sample is nonetheless in close proximity to the type locality of *H. saga* d'Orchymont, 1930(b), while there is no published record of a real *H. gracilis* from any nearby locality; (4) *H. alpicola* and *H. saga* are known to have sometimes identical *COI* sequences (Trizzino et al. 2011a); (5) the margin of the distribution area of *H. alpicola* is more than 300 km away from that collection site (Trizzino et al. 2013a), we assume with reasonable confidence that the said barcode belongs to a *H. saga* specimen and treat it as such herein.

Interspecific genetic distances ranged from 0.0–17.8% (Suppl. material 1). While *Hydraena alpicola* and *H. saga* can obviously not be distinguished by their barcode, the highest divergence was observed for *H. subintegra* and *H. minutissima* as representatives of different main lineages of the same subgenus, *Hydraena* (s.str.).

## Discussion

Due to the recent speciation, it is not surprising that we can barely delineate the species complex nor the individual new species by employing a 2% or 3% species delineation threshold of the mtDNA barcode as originally proposed (Hebert et al. 2003). This aspect is worth noting as it will be impossible to differentiate many young *Hydraena* species by the classical use of DNA barcodes.

Mitochondrial genes, in general and thus standard barcodes, often lag behind in terms of lineage sorting compared to nDNA involved in speciation (e.g. Monaghan et al. 2006, Balke et al. 2013) and this mitochondrial genetic signal can be too unstructured amongst recently radiating species (Meyer and Paulay 2005, Hendrich et al. 2010).

The habitat of *Hydraena dinarica* is quite typical for some highly specialized representatives of the "*Haenydra*" lineage. A similar habitat was also figured for the recently discovered *Hydraena kucinici* Mičetić Stanković & Jäch in the Republic of Macedonia (Mičetić Stanković and Jäch 2012: Figs. 1 and 2). The post-glacial, cold water habitat of *H. dinarica* suggests that it has evolved from a relictual population of a former cold-temperate/sub-polar climate-adapted ancestor related to *H. saga*, which had moved its distribution area southwards during the Pleistocene. Most probably, the new species could not survive in the lowlands of current Montenegro or even in regular surface waters of the same altitude, as reflected by its very restricted habitat and distribution. It can be assumed that the species withstands periods without surface flow (e.g. late summer) hidden in the hyporheic zone.

*Hydraena dinarica* clearly belongs to the "*Haenydra*" lineage (formerly regarded as subgenus by Berthélemy (1986) and Perkins (1997), a clade that was estimated to have occurred in the late Miocene (Tortonian), but however, is not satisfactorily resolved in terms of its phylogenetic position within the genus up to now (Trizzino et al. 2013b). Within the "*Haenydra*" lineage, *H. dinarica*, sp. n. belongs to the *H. gracilis* main clade of about 7–8 Ma age, which is morphologically evident in the articulated aedeagal distal lobe, as well as the low genetic distance from other representatives, based on the 657 bp barcode (Suppl. material 1). The very high morphological similarity, particularly in the articulate aedeagal distal lobe, gonocoxite and female tergite X, as well as the mtDNA data, clearly identify the new species as closely related to *H. saga* and *H. alpicola.* The terminology for the group, to which the latter two and other close congeners belong, is rather confusing and inconsistent. Most recently, it is referred to as *H. saga* complex *sensu* Jäch and Díaz (2017), previously as the *H. emarginata / H. emarginata-saga* complex in a wider sense (Trizzino et al. 2011a, Trizzino et al. 2013a) including the *H. belgica* complex *sensu* Jäch and Díaz (2017), but all of them are not identical to the “*H. saga* complex” as mentioned by Ribera et al. (2011), referring only to the Iberian Peninsula representatives *H. diazi* and *H. fosterorum*. All of the latter are not well supported by DNA data, where *H. saga* and *H. alpicola* (of which mtDNA haplotypes have been reported to be sometimes identical (Trizzino et al. 2011b) usually cluster as a sister sub-clade to the *H. graciles* sub-clade, rather than with the other members of this group (current study, Ribera et al. 2011, Trizzino et al. 2013a). Irrespective of their morphological similarity, the polyphyletic appearance of the eastern (*H. alpicola, H. dinarica, H. saga* and possibly *H. kahleni* for which no genetic data are published) and western (all remaining species of the *H. saga* complex *sensu* Jäch and Díaz (2017)) sub-complexes are not congruent with either definition of such species complex. Redefining the species complexes appears necessary when more genetic information, including nuclear markers, becomes available.

Based on the DNA data available (e.g. current study, Abellán and Ribera 2017, Ribera et al. 2011, Trizzino et al. 2011b), it seems most reasonable to consider the eastern representatives and presumed sister group of the *H. gracilis* complex (Jäch 1995) as a newly-defined *H. saga* complex which, however, is not the purpose of this paper.

Many of these species are known to be highly endemic and all of them are young species, some of the closest relatives, such as *H. saga* and *H. alpicola*, have just split recently during the Pleistocene (about 50,000 years ago, based on data of Trizzino et al. (2013b)). A comparably young age can also be assumed for *H. dinarica*, sp. n. which coincides with the Würm glaciation that covered large parts of Durmitor massif in ice for the last time (Djurović 2009). Ribera et al. (2011) demonstrated a strong non-randomness of the geographic distribution of the species in some "*Haenydra*" clades, meaning phylogenetic distance is congruent to geographic distance of the related species (Fig. 8). This seems to be evident also for *H. dinarica*, sp. n. of which *H. saga* is the geographically closest member of the young species complex and is probably also the closest genetically. Therefore, post-glacial range fragmentation, due to the developing Mediterranean climate with a pronounced arid season, appears to be the most likely scenario for the evolution of *H. dinarica*, sp. n. The obvious ecological preference (or restriction) to its regionally-rare microhabitat with a partly subterranean flow of cold water supports this assumption.

Its rarity, its presumably very limited distribution range and its special habitat association suggest that the new species is particularly vulnerable to climate change and habitat destruction.

## Supplementary Material

C284CBC1-70E4-569A-8BD4-91005ED167DA 10.3897/BDJ.9.e59892.suppl1 Supplementary material 1 Intra- and interspecific *COI* sequence divergence (K2P). Data type genetic divergence table Brief description Specimens sequenced in the field by the use of the *ONTrack* MinION pipeline are indicated by their "H[number]" code. Sequences of Hendrich et al. (2015), Pentinsaari et al. (2014), Ribera (2011), Rulik et al. (2017), Trizzino et al. (2011b; 2013b), are indicated by their respective GenBank accession numbers. File: oo_425124.xlsx https://binary.pensoft.net/file/425124 Hendrik Freitag

E4CEDEDB-2104-5898-A504-DF0316082D8C 10.3897/BDJ.9.e59892.suppl2 Supplementary material 2 Taxon Expedition: the exciting discovery from Durmitor Mountain Data type multimedia File: oo_493321.mp4 https://binary.pensoft.net/file/493321 Clister V. Pangantihon

XML Treatment for Hydraena (Hydraena) biltoni

XML Treatment for Hydraena (Hydraena) minutissima

XML Treatment for Hydraena morio

XML Treatment for Hydraena (Hydraena) nigrita

XML Treatment for Hydraena (Hydraena) spp.

XML Treatment for Hydraena subintegra

XML Treatment for Hydraena (Hydraena) dinarica

## Figures and Tables

**Table 1.** GenBank accession numbers of *Hydraena COI* mtDNA barcode sequences generated in this study.

Species	Specimen	Site	Voucher	Sequencing	ENA	BOLD
*H. dinarica* sp. n.	female	MNE18c	H68	Run1 BC12	MT784148	TXEX049-20
*H. dinarica* sp. n.	male	MNE18c	H67	-	-	-
*H. subintegra*	male	MNE17c	H63	Run1 BC09	MT784149	TXEX051-20
*H. nigrita*	male	MNE17b	H61	Run1 BC08	MT784150	TXEX052-20
*H.* sp. (cf. britteni)	female	MNE10l	H65	Run1 BC10	MT784151	TXEX053-20
*H.* sp.	female	MNE13k	H66	Run1 BC11	MT784152	TXEX054-20
*H. morio*	male	MNE17b	H62	Run2 BC08	MT784154	TXEX055-20
*H. morio*	female	MNE17b	H64	Run2 BC09	MT784155	TXEX056-20
*H. morio*	male	MNE17b	H71	Sanger	MT784156	TXEX057-20
*H. morio*	male	MNE17b	H70	Sanger	MT784157	TXEX058-20
*H. biltoni*	male	MNE21c	H69	Sanger	MT784158	TXEX050-20
*H. minutissima*	male	MNE17b	H60	Run1 BC07	MT784159	TXEX059-20
